# Predicting DNA-Binding Proteins and Binding Residues by Complex Structure Prediction and Application to Human Proteome

**DOI:** 10.1371/journal.pone.0096694

**Published:** 2014-05-02

**Authors:** Huiying Zhao, Jihua Wang, Yaoqi Zhou, Yuedong Yang

**Affiliations:** 1 School of Informatics, Indiana University Purdue University, Indianapolis, Indiana, United States of America; 2 Center for Computational Biology and Bioinformatics, Indiana University School of Medicine, Indianapolis, Indiana, United States of America; 3 QIMR Berghofer Medical Research Institute, Brisbane, Queensland, Australia; 4 Shandong Provincial Key Laboratory of Functional Macromolecular Biophysics, Dezhou University, Dezhou, Shandong, China; 5 Institute for Glycomics and School of Information and Communication Technique, Griffith University, Southport, Queensland, Australia; The University of North Carolina at Charlotte, United States of America

## Abstract

As more and more protein sequences are uncovered from increasingly inexpensive sequencing techniques, an urgent task is to find their functions. This work presents a highly reliable computational technique for predicting DNA-binding function at the level of protein-DNA complex structures, rather than low-resolution two-state prediction of DNA-binding as most existing techniques do. The method first predicts protein-DNA complex structure by utilizing the template-based structure prediction technique HHblits, followed by binding affinity prediction based on a knowledge-based energy function (Distance-scaled finite ideal-gas reference state for protein-DNA interactions). A leave-one-out cross validation of the method based on 179 DNA-binding and 3797 non-binding protein domains achieves a Matthews correlation coefficient (MCC) of 0.77 with high precision (94%) and high sensitivity (65%). We further found 51% sensitivity for 82 newly determined structures of DNA-binding proteins and 56% sensitivity for the human proteome. In addition, the method provides a reasonably accurate prediction of DNA-binding residues in proteins based on predicted DNA-binding complex structures. Its application to human proteome leads to more than 300 novel DNA-binding proteins; some of these predicted structures were validated by known structures of homologous proteins in APO forms. The method [SPOT-Seq (DNA)] is available as an on-line server at http://sparks-lab.org.

## Introduction

The completion of thousands of proteome projects has led to an explosive increase in number of proteins with unknown functions. The comprehensive Uniprot database [1] contains 10^7^ protein sequences and, yet, less than 5% of these sequences have annotated functions from Gene Ontology Annotation database [2]. This gap between the number of sequences and the number of sequences with annotations is widening rapidly as inexpensive and more efficient next generation sequencing techniques become available. Experimentally identifying function of millions of proteins is obviously impractical. Thus, it is necessary to develop effective bioinformatics tools for initial functional annotations.

One important function of proteins is DNA-binding that plays an essential role in transcription regulation, replication, packaging, repair and rearrangement. Function prediction of DNA-binding can be classified into three levels of resolution (low, medium and high). A low-resolution function prediction is a simple two-state prediction whether or not a protein binds to DNA. A medium-resolution function prediction is to predict the region in a protein that binds with DNA (DNA-binding residues or DNA-binding interface regions). A high-resolution function prediction is to predict the complex structure between DNA and a target protein of unknown function.

Most existing methods have been focused on two-state (low resolution) prediction [3]–[20] and prediction of binding residues (medium resolution) [6], [9], [21]–[45]. The majority of these techniques are based on machine-learning techniques ranging from neutral networks, random forest, decision trees to support vector machines that are trained on the features derived from sequence (sequence-based) and structure (structure-based). A structure-based technique attempts to infer functions from known protein structures. Both sequence-based [4], [6], [10], [12], [14], [15], [18], [20] and structure-based [3], [7]–[9], [13], [16], [17], [19] prediction of DNA-binding proteins were developed. The same is true for binding residue prediction (Sequence-based [6], [22], [25], [31], [33], [34], [36], [37], [39], [41], [42], [45] and structure-based [9], [21], [23], [24], [26]–[28], [32], [35], [38], [43]).

An alternative approach to above machine-learning techniques is to take advantage of known protein-DNA complex structures. This can be accomplished by structural comparison between a DNA-binding template and a target protein structure [5], [11], [29], [30]. For example, we demonstrated that a size-independent, structural alignment method SPalign makes a significant improvement over several other commonly used tools for locating functionally similar structures [11]. If the structure of a target protein is unknown, homology modeling [40], [46] has been employed. Gao and Skolnick further illustrated the importance of combining structure prediction (through structural alignment [47] or threading [48]) with binding prediction for detecting DNA-binding proteins. One important aspect of this approach is its ability to predict the complex structure between a target protein and template DNA. This high-resolution function prediction at atomic details allows an improved understanding of binding mechanism and an integration with prediction of DNA-binding proteins and DNA-binding residues.

This work focuses on improving the high-resolution function prediction. The DBD-Threader method developed by Gao and Skolnick [48] first employed the threading technique called PROSPECTOR [49] to predict structures based on known DNA-binding domains. Confidently predicted complex structures are then confirmed for DNA-binding by utilizing a pairwise knowledge-based, contact energy function [47]. The method has achieved the Matthews correlation coefficient (MCC) of 0.68 for the two-state prediction of DNA-binding proteins by using a database of 179 DNA-binding domains (DB179) and 3797 non-DNA-binding domains (DB3797).

In this work, we approach this function prediction problem with different methods for protein-structure prediction and binding affinity prediction. Instead of a contact-based energy function employed in DBD-Threader [48], we employed a statistical energy function based on a distance-scaled ideal-gas reference state (DFIRE) [50] extended for protein-DNA interactions [51]–[53]. This DDNA energy function was found useful in developing a highly accurate structure-based technique called SPOT-Struc (DNA) that achieved the MCC value of 0.76 for the same database of DB179 and NB3797, employed by DBD-Threader. In addition to energy functions, we examined two fold-recognition techniques to enable a sequence-based prediction as DBD-Threader. One is a method based on hidden Markov model (HHM) called HHblits [54]. The other is our in-house built technique called SPARKS-X [55]. Both methods are among the top performers in critical assessment of protein structure prediction techniques (CASP 9) [55], [56]. This development of SPOT-Seq for DNA-binding proteins was inspired by the success of prediction of RNA-binding proteins [57] by integrating SPARKS for structure prediction and DFIRE for protein-RNA binding prediction [58] and its successful application to human proteome [59].

SPOT-Seq for DBPs was applied to DB179 and NB3797 and achieved a MCC value of 0.77 for DBP prediction by combining HHblits with the DDNA3 energy function (leave-one-out). The method was further tested on newly determined DBPs (positive set), RNA-binding proteins (negative set), and the human proteome as well as SCOP folds that host both DNA and non-DNA binding proteins. All results confirmed that the method is highly sensitive (>50%) and its performance is consistent in various tests. More than 300 novel DBPs were found in human proteome. For binding residue prediction, the average MCC values are 0.55 for 116 predicted DBPs in DB179 and 0.64 for 42 predicted DBPs in newly solved structures (DB82).

## Materials and Methods

### Gao-Skolnick domain datasets (DB179 and NB3797)

Gao and Skolnick complied two datasets that contain 179 DNA-binding protein domains and 3797 non-DNA binding protein domains [47]. They were obtained by collecting the proteins with a resolution of 3 Å or better, a minimum length of 40 amino acid residues per protein and at least 6 base pairs of DNA and five residues interacting with DNA. The redundant data between two sets were excluded by using 35% sequence identity cutoff. DB179 is used as a template library in this work.

### Test set of RNA-binding proteins (RB174)

RB174 is a dataset made of 174 high-resolution RNA-binding proteins (whole chains), collected by us in developing SPOT-Seq (RNA) based on a 25% cutoff. We employed RB174 to examine if the proposed method can separate DNA-binding proteins from RNA-binding proteins.

### Independent test dataset (DB82)

An independent test set was built by obtaining the DNA-binding proteins released after December 2009. The protein chains were divided into SCOP domains, and the redundant data was removed by using sequence identity cutoff of 30%. We further excluded the proteins that have sequence identity higher than 30% with any proteins in DB179. Finally, we generated an independent test dataset with 82 protein domains (chains if SCOP domains were not available).

### Function prediction protocol

The prediction protocol proposed here is the same as SPOT-seq (RNA) developed by us [57], except that 1) the template library is made of known protein-DNA complex structures and 2) HHblits [54], in addition to SPARKS-X [55], is used in structure prediction. Briefly, HHblits (or SPARKS-X) is employed to match a target sequence to template structures in the template library. If a significant match is found based on a matching probability (HHblits) [or Z-score (SPARKS)], the top matched template(s) are then utilized to model protein-DNA complex structure(s) by copying the query sequence to the template complex structure(s) according to the alignment result while keeping the template DNA intact. The complex-structure models are then employed to estimate the binding affinity between the target protein (main-chain only) and the template DNA by utilizing DDNA3 [53]. The target protein is classified as DNA-binding if the binding affinity is higher than a threshold. Thus, there are only two parameters to be optimized: sequence-structure matching score (or Z-score for SPARKS) and the binding energy value.

### Performance evaluation

The performance of the method is evaluated by sensitivity [SN  =  TP/(TP + FN)], precision [PR  =  TP/(TP + FP)], specificity [SP  =  TN/(TN + FP)], accuracy [AC  =  (TP + TN)/(TP + FN + TN + FP)], and Matthews correlation coefficient (MCC) given by 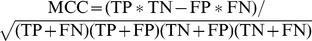. Here, TP, TN, FP, and FN refer to true positives, true negatives, false positives and false negatives, respectively. A MCC value provides an overall assessment of the method performance with 1 for perfect agreement and 0 for random prediction. One should note that sensitivity can also be called as coverage of true positive prediction while precision is fraction of corrected predictions in all positive predictions.

### HHblits

HHblits [54] is a fold-recognition technique that extracts homologous sequences of targets from template library by Hidden-Markov models (HMM). The HHM matrices of targets and templates are built by searching against the Uniprot database. The probability of a match is calculated by comparing the HMM matrix of a target to the HMM matrix of a template. We define a target sequence as a DBP if the probability of a match is higher than a threshold. The threshold is optimized by maximizing the MCC value. HHblits was downloaded from http://toolkit.tuebingen.mpg.de/HHblits. Default parameters were utilized in structure prediction.

## Results

### Low-resolution function prediction (binding or not binding)

#### 1. Leave-one-out cross validation (Gao-Skolnick datasets)

**Figure 1:**
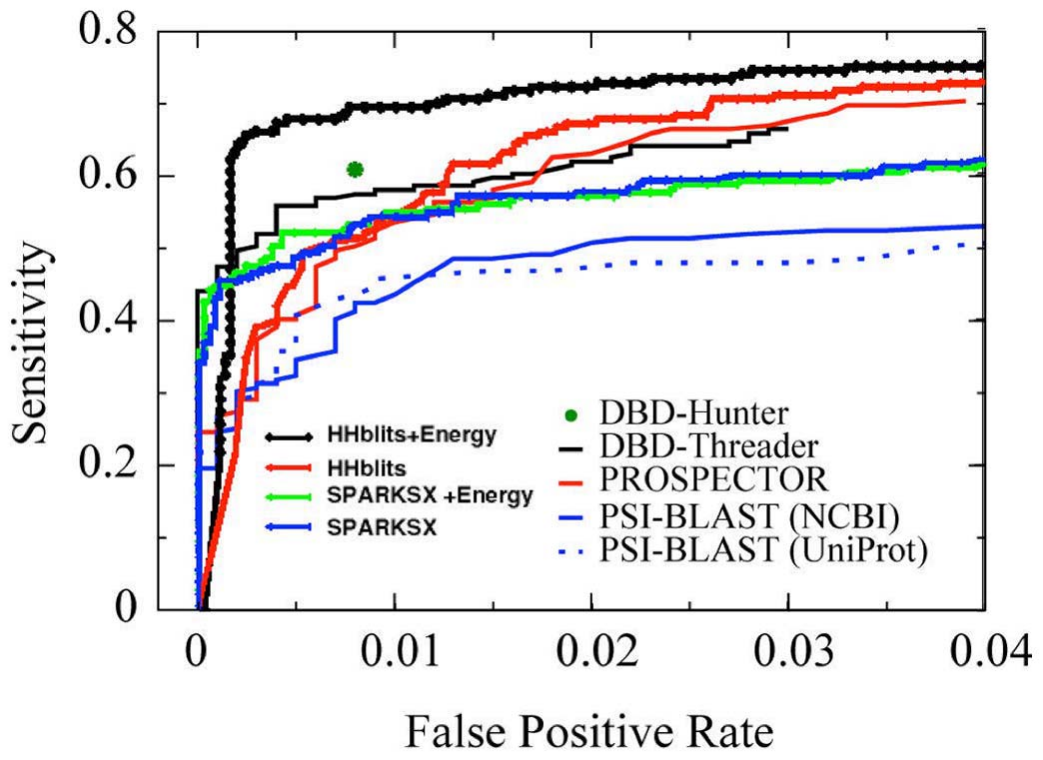
Performance of various methods for DNA-binding protein prediction (leave-one-out cross validation).

A leave-one-out cross validation is conducted by removing all templates with >30% sequence identity to the target. The results were obtained by taking one chain sequence from DB179 or NB3797 and predicting whether it binds or does not bind to DNA. Figure 1 and Table 1 compared the methods based on known protein structures (DBD-Hunter [48], DDNA3 [53], and DDNA3O [53]), purely sequence-based (PSI-BLAST (NCBI) [48], PSI-BLAST(uniprot) [48]), sequence and template-structure-based (PROSPECTOR [48], HHblits, SPARKS-X), and incorporation of an energy function (DBD-Threader [48], SPARKS-X+Energy, HHblits+Energy). For sequence-based fold/homology-recognition techniques, SPARKS-X yields the highest MCC value (0.647), followed by HHblits (0.639), PROSPECTOR (0.609), and PSI-BLAST (0.553 or 0.540). Adding the energy function to fold recognition leads to a small improvement over SPARKS-X (MCC from 0.647 to 0.652) but a large improvement over PROSPECTOR (MCC from 0.609 to 0.681) and over HHblits (MCC from 0.639 to 0.771). In particular, the best performing HHblits + Energy leads to a sensitivity of 65% and precision of 94%. Such performance is even better than the best structure-based technique (DDNA3O) with a MCC value of 0.76 (0.73 without TM-Score dependent optimization). Because combining HHblits with our energy function leads to a significantly improved method than combining SPARKS and the energy function, we mainly focus on the former here and below, unless indicated otherwise.

**Table 1:** Performance of various methods for predicting DNA-binding proteins.

Methods	SN(%)^a^	PR(%)^a^	SP(%)^a^	ACC a	MCC a
**Structure-based^b^**					
DBD-Hunter^c^	61	79	92	-	0.681
DDNA3^d^	60	91	99	98	0.73
DDNA3O^d^	64	93	99.8	-	0.76
**Sequence-based**					
PSI-BLAST(NCBI)^ e^	49	64	87	-	0.540
PSI-BLAST(Uniprot)^e^	43	75	93	-	0.553
**Sequence and template-structure based**					
Prospector^e^	53	74	91	-	0.609
HHblits	61	69	99	97	0.639
SPARKS-X	45	95	99	97	0.647
**Sequence and template-structure based, plus energy scoring**					
SPARKS-X+Energy	53	84	99	97	0.652
DBD-Threader^e^	56	86	96	-	0.680
HHblits+Energy	65	94	99	98	0.771

a SN, sensitivity; PR, precision; SP, specificity; ACC, accuracy; MCC, Matthews correlation coefficient.^ b^Methods based on known protein structures. ^c^From Ref. [47] ^d^from Ref. [53]. ^e^from Ref. [48].

#### 2. Separating DNA-binding from non-DNA-binding in the same SCOP fold

One crucial test of a method for predicting DNA-binding function is to examine whether or not it can classify DBPs from non-DBPs within the same structural fold. We analyzed 18 SCOP folds shared by DNA-binding and non-DNA-binding proteins. As shown in Table 2, after incorporating the DDNA energy function for DBP prediction, the number of true positives increases from 57 to 61 and false positives decreases from 24 to 4. Thus, removal of false positives is the key factor for large improvement when an energy function is employed.

**Table 2:** Detecting DBPs in 18 structural folds shared by DNA-binding and non-binding proteins.

*Fold*	*Dataset* *(bd/nb)*	*HHblits* *(bd/nb)*	*HHblits+Energy* *(bd/nb)*
A.38	5/1	5/0	5/0
A.74	4/10	1/2	1/2
C.52	14/4	3/0	4/0
A.4	50/11	23/0	25/0
A.6	2/2	2/0	2/0
C.66	4/19	4/15	3/0
C.62	2/10	2/0	2/0
G.39	2/12	1/0	1/0
C.37	5/87	2/5	2/0
D.151	2/2	2/2	1/2
A.60	7/1	4/0	5/0
D.95	6/1	2/0	3/0
C.55	8/35	2/0	1/0
B.82	1/37	0/0	1/0
C.53	1/5	1/0	1/0
H.1	5/43	2/0	2/0
D.129	3/13	0/0	1/0
D.218	1/8	1/0	1/0
Total	122/301	57/24	61/4

### Medium-resolution function prediction (DNA-binding residues)

The complex structures predicted from our method allow us to infer amino-acid residues involved in DNA-binding. We define an amino-acid residue as a DNA-binding residue if any heavy atoms of the residue are less than 4.5Å away from any heavy atoms of a DNA base as in [47]. The accuracy of binding-residue prediction is examined on 116 true positive predictions from DB179. The final values of MCC, sensitivity, and precision of the prediction averaged over 116 proteins are 0.55, 57%, 66%, respectively. A similar, average MCC value (0.54) was obtained if SPARKS-X was used to perform structure prediction.

**Figure 2:**
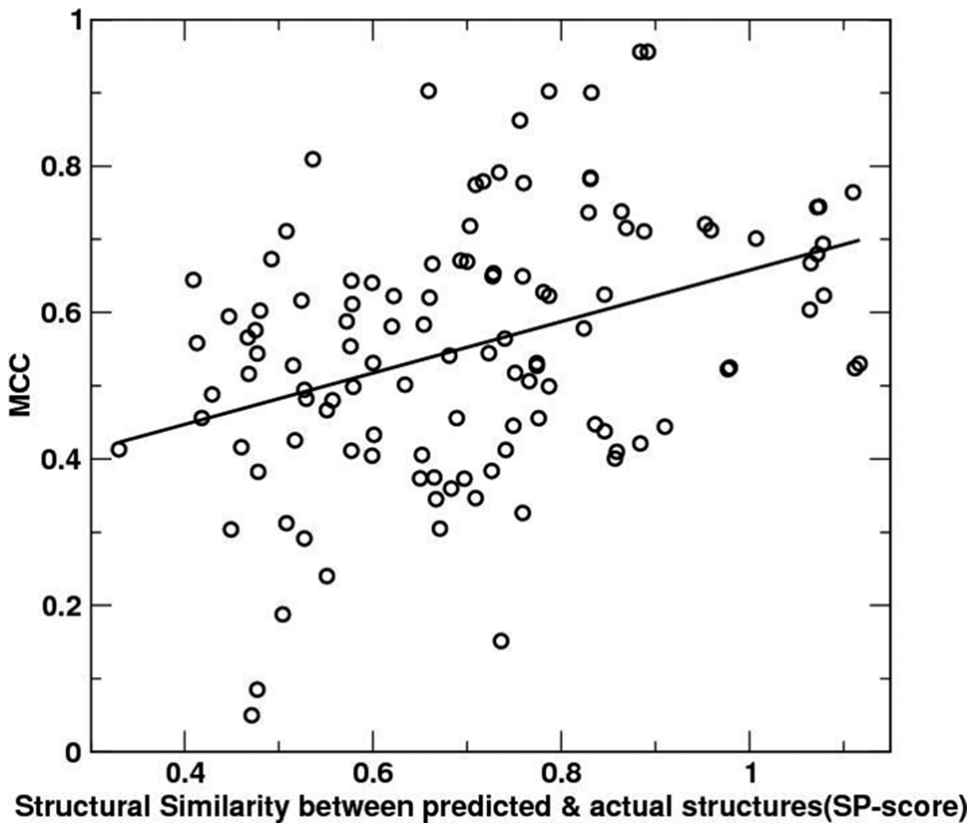
Matthews correlation coefficient for predicted binding residues versus the structural similarity SP-score between predicted and known structures of 116 targets. The correlation coefficient is 0.38.

The quality of predicted binding residues is directly related to the quality of predicted structures as expected. Figure 2 shows the MCC for binding residue prediction as a function of predicted structural accuracy according to structural similarity between predicted and actual structures by SPscore. There is a trend that the higher accuracy for predicted structures, the higher the MCC value is. The correlation coefficient is 0.38. We noticed that there are a few cases of highly accurate structures but with poorly predicted binding regions (low MCC values). In those cases, accurate structures were limited to non-binding regions.

### High-resolution function prediction (complex structure prediction)

The quality of predicted DNA-binding complex structures was examined by the structural alignment SPalign [11] that compares native structures and predicted structures based on a size-independent structural similarity score called SPscore. Two structures are considered as in the same fold if SPscore>0.5 [11]. For 116 correctly predicted targets, the average SPscore is 0.65. The structure similarity can also be evaluated by the fraction of aligned residues with a root mean-squared distance (RMSD) between two compared structures less than 4Å. We found that the medium value is 67%.

**Figure 3:**
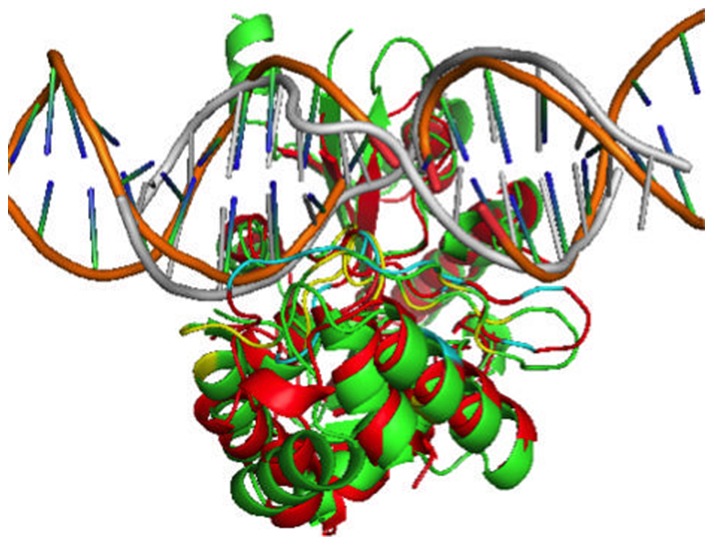
Comparison of predicted (red) and native structures (green) of target 1yfjD (DAM). Native structure and DNA are represented by green and orange, respectively. The predicted structure and DNA are denoted by color red and grey. The predicted binding sites and native binding sites are in cyan and yellow colors, respectively.

As an example, Figure 3 compared predicted binding sites with native binding sites, and the predicted structure with the native structure for the target (bacteriophage T4 DNA-adenine methyltransferase, T4-dam, PDB# 1yfjd,). The sequence identity between the target and the template (2g1pa, dam) is 24%. Predicted (light grey) and actual (orange) DNAs overlap with each other very well when protein structures are aligned. Predicted binding sites (cyan) are also consistent with the native binding region (yellow) with an MCC value of 0.60.

### Independent tests

#### 1. Negative set -Separating RNA-binding proteins from DNA-binding proteins

As RNA-protein interactions share some similar characteristics with DNA-binding proteins (both are positively charged, for example), it is important to examine if the proposed method can separate DBPs from RBPs. We tested the HHblits+energy method with the thresholds optimized by DB179+NB3797 datasets on the RBP dataset (RB174). It predicts 5 proteins as DBPs. Two of the five (1zbib and 1hysa) are highly homologous (sequence identity >70%) to the templates (1zblb and 1r0aa, respectively). Proteins in 1zbib (*Bacillus halodurans* RNase H catalytic domain) and 1r0aa (HIV-1 reverse transcriptase) are related to dual RNA- and DNA-binding functions. 1zbib is a complex structure between *Bacillus halodurans* RNase H catalytic domain and 12mer RNA/DNA hybrid. HIV-1 reverse transcriptase in 1r0aa is a RNA-dependent DNA polymerase. Two of the three remaining proteins (2qk9a and 1ooaa) are also annotated as DNA-binding. 2qk9a is Human RNase H catalytic domain binding with both RNA and DNA [60] and 1ooaa contains Rel homology domain (RHD) and DNA binding site [61]. The only remaining protein (PDB ID 2jlua) is dengue virus 4Ns3 helicase in complex with ssrna [62]. This helicase was found to function on both RNA and DNA templates [63]. Thus, there is zero false positive in DNA-binding prediction.

#### 2. Positive set –Newly determined complex structures (DB82)

We tested the performance of SPOT-Seq (DNA) by utilizing 82 newly determined protein-DNA complex structures. SPOT-Seq correctly predicted 42 (51%) as DBPs based on the same thresholds obtained from the leave-one-out (matching probability of 84% and energy threshold of −8.6). The average MCC value for predicted binding residues of these 42 proteins is 0.64. The average structural similarity between predicted and actual structures is 0.73 based on SPalign [11]. As shown in Table 3, the sensitivity of two-state RBP prediction decreases from 65% for DB179 to 51% for this smaller DB82 test set while the average MCC value of binding residue prediction increases from 0.55 to 0.64 and the average structural similarity between predicted and actual structures increases from 0.65 to 0.73 according to SPscore.

**Table 3:** Performance of SPOT-Seq on prediction of DNA-binding proteins at three resolution levels.

Measure	DB179/NB3797	DB82
**Two-state prediction**		
MCC	0.77	-
Accuracy	98%	-
Precision	93%	-
Sensitivity	65%	51%
**Binding residue prediction**		
MCC	0.52	0.64
Accuracy	88%	93%
Precision	63%	67%
Sensitivity	55%	69%
**Structure prediction**		
SPscore	0.65	0.73
RMSD(<4 Å)	67%	68%

### Application to Human Proteome

Our approach was utilized to detect DBPs from human proteome. The human proteome with 20270 proteins was downloaded in 2010 from Uniprot [1]. We obtained annotations of human proteins from Gene Ontology (GO) [64]. The following DNA-related GO keywords are employed for defining an annotated DBP: “DNA binding”, “transcription factor” and others (“DNA replication”, “DNA repair”, “DNA recombination”, “DNA helicase activity”). Such definition leads to 2883 annotated DBPs in 20270 proteins. The number of proteins in each category is listed in Table 4.

**Table 4:** Number of annotated and predicted DBPs in the human proteome.

*Function*	*Number of Annotated*	*Number of Predicted*	*Recovery rate (Sensitivity)*
Transcription factor	1459	837	61%
DNA binding	1239	763	62%
DNA repair	91	6	7%
DNA recombination	10	1	1%
DNA replication	51	3	6%
DNA-related biological process	33	2	6%
Total	2883	1612	56%

Our sequence-based technique (HHblits+Energy) predicted 1975 out of 20270 proteins as DBPs. Among 1975 proteins, the majority (1612, 82%) predicted DBPs were annotated as DBPs according our definition above. The recovery (or sensitivity) of our method is 56% (1612/2883) annotated DBPs. Remaining 363 predicted DBPs were not annotated as DBPs according to Gene Ontology, in which 259 proteins were annotated with other functions and 104 proteins with no annotation in Gene Ontology. The recovery rate (sensitivity) of our prediction for each keyword is shown in Table 4. They are 61% for transcription factors and 62% for DNA binding but low for other keywords.

We examined 363 newly discovered DBPs in more details. We found that 23 of these predicted new DBPs (Table 5) have homologs (>60% sequence identity) with known experimental structures in predicted structural regions. The majority of these structures (21/23) are either in a monomeric form or in complex with itself or other proteins. Interestingly, two of 23 structures contain DNA, a direct confirmation of their DNA binding capability. They are Uracil-DNA glycosylase that involves with DNA repair [65] and steroid hormone receptor ERR2 that has ligand-activated sequence-specific DNA binding RNA polymerase II transcription factor activity [66]. These two proteins were not annotated in GO as DNA binding. Predicted structures for these 23 proteins are highly similar to the structures of their corresponding homologs in the majority of cases (16/23 or 70% with SPscore ≥0.5, an indication of same structural fold). For those predicted structures with <0.5 SPscore with corresponding structures of their homologs, the majority (5/7) has a matching region of <60 residues. Such small matching region is more likely to have binding induced conformational change. Seven in 23 proteins are Cyclin proteins that are involved in regulation of cell cycles.

**Table 5:** Predicted DBPs whose homologs have experimentally determined 3-dimensional structures.

Uniprot ID	Name	TPL	Homo chains	SP-score	SeqID (%)	L_match_
P13051	Uracil-DNA glycosylase	4skne	1emha	1.329	98.7	224
P24855	Deoxyribonuclease-1	2dnja	4awna	1.021	97.3	99
O75909	Cyclin-K(DNA-dependent_transcription_regulation)	1c9be	2i53a	0.853	75.6	76
P38919	Eukaryotic initiation factor 4A-III (RNA_helicase)	2p6ra	2j0qa	0.808	91.0	114
O95718	Steroid hormone receptor ERR2 (DNA binding)	1kb4a	1lo1a	0.799	93.4	86
P30281	G1/S-specific cyclin-D3	1c9be	3g33b	0.773	82.1	63
P20248	Cyclin-A2	1c9be	2wipb	0.773	80.2	64
P24385	G1/S-specific cyclin-D1	1c9be	2w96a	0.765	79.1	63
P14635	G2/mitotic-specific cyclin-B1	1c9be	2b9ra	0.746	80.7	116
P24863	Cyclin-C	1c9be	3rgfb	0.742	76.3	115
P51946	Cyclin-H	1c9be	1jkwa	0.733	73.6	56
Q9UMR2	ATP-dependent RNA helicase DDX19B	2p6ra)	3ewsa	0.731	83.9	223
O60942	Mrna-capping enzyme (GTP binding)	2owoa	3s24a	0.615	75.0	87
Q9UNQ2	Probable dimethyladenosine transferase (rrna binding)	1dctb	1zq9a	0.562	82.4	107
Q9NRR6	72 kda inositol polyphosphate 5-phosphatase	1dewb	2xswa	0.539	75.3	66
P32019	Type II inositol 1,4,5-trisphosphate 5-phosphatase	1dewb	3n9va	0.500	81.3	41
Q96LA8	Protein arginine N-methyltransferase 6	2ibsa	4hc4a	0.492	81.6	98
Q96LI5	CCR4-NOT transcription complex subunit 6-like (Nuclease)	1dewb	3ngna	0.479	75.0	38
Q96AZ6	Interferon-stimulated gene 20 kda protein (Ribonuclease)	2pyjb	1wlja	0.472	78.0	53
P09234	U1 small nuclear ribonucleoprotein C (mrna binding)	2i13a	2vrda	0.363	75.4	33
Q16281	Cyclic nucleotide-gated cation channel alpha-3	1cgpa	3swya	0.342	67.7	40
Q9NRK6	ATP-binding cassette sub-family B member 10, mitochondrial	2o8db	4ayta	0.310	76.2	140
Q9BW91	ADP-ribose pyrophosphatase, mitochondrial	1rrqa	1q33a	0.207	72.6	57

## Discussion

In this paper, we have developed a sequence-based method that predicts DNA-binding proteins and their complex structures with DNA based on existing protein-DNA complex structures. The method achieved a MCC value of 0.77 that is higher than the best structure-based technique (DDNA3O). The method achieved >50% sensitivity in the independent test set of newly solved protein-DNA complex structures and 56% for human proteome. The method also has a 94% precision in leave-one-out cross validation. Such high precision is confirmed by the fact that 82% predicted DBPs in human proteome were annotated as DBPs. Because template-based methods depend on appropriate templates in the database, a limited number of templates made the methods with high precision but relatively low sensitivity (coverage). An improved fold recognition method is critical for further increasing the sensitivity.

One interesting observation is that combining HHblits with DDNA energy function, rather than combining SPARKS-X with DDNA energy function, yields the best performance, despite the fact that SPARKS-X alone produces a higher MCC value (0.647) than HHblits alone (0.639). This suggests a cancellation of errors where over-prediction made by HHblits is corrected by the energy function.

This work also reveals that DBPs are easier to identify than RBPs. The sensitivity for DBP prediction is 56% in human proteome, compared to only 43% for RBP prediction [59]. Moreover, <400 new DBPs are discovered, compared to >2000 new RBPs in human proteome. This is mainly because RNA structures are much more complex and diverse than that of DNA. Moreover, RNA-binding proteins are not as well studied as DNA-binding proteins.

Here, the analysis of DNA-binding function is mostly done with GO annotations. We found that the GO annotation is not complete for some proteins. The known DNA-binding protein such as Uracil-DNA glycosylase [65] in Table 5 was not included in GO annotations but were annotated in Uniprot. We further found that 49 (13%) out of 363 predicted novel DBPs are annotated as DBPs in the DAVID database [67]. This further reduces the number of novel predicted DBPs to 314. Some of these novel DNA-binding proteins in Table 5 are nucleases and helicases that could operate on both DNA and RNA (e.g. CCR4 and DDX19B in Table 5). Others are less obvious for their putative DNA-interacting capability and warrants further investigations.

Finally, it is worthy to mention that the template-based approach presented here for DBP prediction is reasonably fast. It takes about a month on a single processor PC (or 2 days with a 16-core server) to scan all proteins in human proteome. The method [SPOT-Seq (DNA)] is available on line as a server at http://sparks-lab.org.
